# Cladribine treatment of multiple sclerosis is associated with depletion of memory B cells

**DOI:** 10.1007/s00415-018-8830-y

**Published:** 2018-03-17

**Authors:** Bryan Ceronie, Benjamin M. Jacobs, David Baker, Nicolas Dubuisson, Zhifeng Mao, Francesca Ammoscato, Helen Lock, Hilary J. Longhurst, Gavin Giovannoni, Klaus Schmierer

**Affiliations:** 1 Blizard Institute, Barts and the London School of Medicine and Dentistry, Queen Mary University of London, 4 Newark Street, London, E1 2AT UK; 2 Haematology Unit, The Royal London Hospital, Barts Health NHS Trust, London, UK; 3 Emergency Care and Acute Medicine Clinical Academic Group Neuroscience, The Royal London Hospital, Barts Health NHS Trust, London, UK

**Keywords:** B cell, Cladribine, Disease-modifying treatment, Deoxycytidine kinase, Multiple sclerosis, Memory B cells

## Abstract

**Background:**

The mechanism of action of oral cladribine, recently licensed for relapsing multiple sclerosis, is unknown.

**Objective:**

To determine whether cladribine depletes memory B cells consistent with our recent hypothesis that effective, disease-modifying treatments act by physical/functional depletion of memory B cells.

**Methods:**

A cross-sectional study examined 40 people with multiple sclerosis at the end of the first cycle of alemtuzumab or injectable cladribine. The relative proportions and absolute numbers of peripheral blood B lymphocyte subsets were measured using flow cytometry. Cell-subtype expression of genes involved in cladribine metabolism was examined from data in public repositories.

**Results:**

Cladribine markedly depleted class-switched and unswitched memory B cells to levels comparable with alemtuzumab, but without the associated initial lymphopenia. CD3^+^ T cell depletion was modest. The mRNA expression of metabolism genes varied between lymphocyte subsets. A high ratio of deoxycytidine kinase to group I cytosolic 5′ nucleotidase expression was present in B cells and was particularly high in mature, memory and notably germinal centre B cells, but not plasma cells.

**Conclusions:**

Selective B cell cytotoxicity coupled with slow repopulation kinetics results in long-term, memory B cell depletion by cladribine. These may offer a new target, possibly with potential biomarker activity, for future drug development.

**Electronic supplementary material:**

The online version of this article (10.1007/s00415-018-8830-y) contains supplementary material, which is available to authorized users.

## Introduction

Multiple sclerosis (MS) is a chronic-inflammatory disorder of the central nervous system leading to accumulating disability. Historically, myelin-directed, CD4^+^, Th17^+^ cells have been considered to be the important mediators of the inflammatory process [1]. This is largely based on animal experimental autoimmune encephalitis models where T cell-mediated responses are key to pathogenesis [2]. However, suppression of CD4^+^, Th17^+^ cells has only produced modest benefits in clinical trials in MS [3, 4]. Therefore, other mechanisms may play a crucial role in the disease process.

Whilst animal models and in vitro studies in MS provide circumstantial evidence for the involvement of particular pathways, positive and properly conducted, negative trials provide invaluable information for understanding the cellular mechanisms that drive disease activity. Alemtuzumab, a CD52, T and B cell depleting antibody [5], ocrelizumab, a CD20-B cell depleting antibody [6] and cladribine, a lymphocyte depleting agent [7], are immune-reconstitution therapies that are amongst the most efficacious treatments for MS and can provide long-term benefit from few treatment cycles [4, 8, 9]. However, there is no clear consensus on their mode of action, particularly for cladribine [7, 10].

It is well known that lymphocytes produce many secreted proteins, rapidly proliferate in response to infections and rely on both de novo and importantly salvage pathways for nucleotide synthesis. To accommodate this requirement, lymphocytes express high levels of adenosine deaminase (ADA) protein [11, 12]. This prevents the accumulation of cytotoxic levels of deoxyadenosine triphosphate, by catalysing the deamination of adenosines to inosines [12]. Cladribine is a chlorinated analogue of deoxyadenosine that is partially resistant to ADA [12, 13]. This is phosphorylated by deoxycytidine kinase (DCK) to create lymphocyte cytotoxicity [12]. This action is countered by cytosolic 5′ nucleotidase (5′NT) enzymatic activity that dephosphorylates phosphorylated-cladribine. Lymphocytes and lymphocytic cancers, including hairy cell leukaemia for which parenteral cladribine is licensed, have a high DCK-to-5′NT ratio relative to other cell types and are susceptible to cladribine-induced cytotoxicity [12].

An oral-cladribine prodrug was shown to be very effective at controlling relapsing MS [7, 8]. This was first licensed in 2011, but was later withdrawn when regulators requested more studies to address issues related to severe lymphopenia (25.6% (*n* = 110/430) grade 3/4 found in year 2) and cancer seen in the pivotal trial [7, 10]. However, we demonstrated that the short-term cancer risk of oral cladribine was no greater than for any of the licensed disease modifying treatments (DMT) [14], and instigated a compassionate-use programme using generic, subcutaneous cladribine that was dose-adapted to limit severe lymphopenia [15]. This and probably the licensing of alemtuzumab, which induces significantly more lymphopenia and side-effects than cladribine [5, 7, 16], prompted re-submission of cladribine tablets to the regulators. These were licensed in Europe for the treatment of relapsing MS [10].

Although, cladribine is considered to be a T and B cell inhibitor, emphasis has been placed on T cell inhibition as a mechanism of action [10, 17]. However, immunophenotyping data demonstrated that effective doses of oral cladribine induced only about a 20–30% depletion of CD8^+^ T cells and about a 40–45% depletion of CD4^+^ T cells within 12 months that was reflected by comparable memory T cell depletion, but induced marked (80–85%) CD19^+^ B cell depletion [16]. Alemtuzumab induced a similar, but more transient depletion, of CD19^+^ B cells [18] again focussing attention towards the long-term depletion of T cells as a mechanism of action [5]. However, analysis of CD19^+^ subpopulations in alemtuzumab-treated people with MS (PwMS) demonstrated that it was a composite response of enhanced immature and mature B cell hyper-repopulation that masked a substantial and sustained depletion of memory B cells [18]. Importantly, we identified that memory B cells were depleted by all DMT that inhibit MS, in a manner reflecting their efficacy in controlling relapsing MS [4, 19]. We therefore hypothesised that given the high efficacy of cladribine [7, 8], it too would deplete memory B cells and that public gene expression databases would contain data on purine salvage pathways genes that may explain its mechanism of action.

## Materials and methods

### Ethical review

The study was approved by the Health and Social Care Research Ethics Committee B and the Health Research Authority, UK. People were recruited following informed consent. Purchased blood samples were collected with informed consent and did not require additional ethical review.

### Participants

Participants (Supplementary Table S1) were diagnosed with MS based on the revised McDonald criteria. Subjects provided demographic details and drug history at the time of informed consent and completed an online Extended Disability Status Scale (EDSS) (https://edss.clinicspeak.com). Inclusion criteria are diagnosis of MS; aged 18–65 years and either on alemtuzumab or cladribine therapy or drug-naive (MS controls) undergoing investigations to start treatment. Healthy control volunteers also provided blood samples with informed consent. People were excluded if they could not give valid consent or comply with the study requirements.

### Treatments

PwMS were treated with 12 mg/day alemtuzumab for 5 days at month 1 and 12 mg/day for 3 days at month 13 [5]. Subcutaneous cladribine was used off-label on compassionate grounds. This consisted of 3 × 10 mg adjusted for weight (4 × 10 mg if > 90 kg) at month 0 and repeated 4 weeks later and further dose-adjusted from 0 to 4, 10 mg doses depending on subject weight and level of lymphopenia with > 1.0 × 10^9^ cells/L receiving three doses, 0.8 to  < 1.0 × 10^9^ cells/L receiving two doses; 0.5 to  < 0.8 × 10^9^ cells/L receiving one dose and those with < 0.5 × 10^9^ cells/L receiving no additional doses [15].

### Study design

A cross-sectional study was performed on PwMS attending the Royal London Hospital, for routine blood monitoring as part of their standard of care. Samples were collected from healthy controls; drug-naïve PwMS and PwMS at their end of year blood test, prior to their second cycle of treatment.

### Lymphopenia assessment

The level of lymphopenia was assessed via standard haematology laboratory measures. One 1-year data was extracted from the pivotal CLARITY dataset of oral cladribine [4–5 daily dosing of 10–20 mg tablets (4–8 mg/day generic cladribine equivalent) week 1 and 5] of the group that were immunophenotyped (*n* = 309) supplied by the European Medicines Agency (EMA) under a Freedom of Information request [19]. The data from the 3.5 and 5.25 mg/kg groups [9] were merged as they had both received identical dosing up to 9 weeks. Analysis was restricted to data, where baseline 5- and 9-week data were all available. This was compared and contrasted with anonymous audits of people treated with subcutaneous, generic cladribine (3–4, 10 mg subcutaneous cladribine) doses at week 1 and week 5 or 5 daily 12-mg doses of alemtuzumab. The nadir at week 4 or 9 was selected for cladribine and the depletion at week 5 was analysed for alemtuzumab. The grade of lymphopenia was based on standardised definitions of the Common Terminology Criteria for adverse events V4, National Cancer Institute, Bethesda. Differences in the frequency of severe lymphopenic events were estimated using Chi-squared analysis.

### Immunostaining

Anonymised heparinised venous blood samples were collected and cells were labelled for 1 h with mouse anti-human antibody–fluorophore conjugates: CD3-PE (T cells), CD10-PECy7, CD19-PECy5, CD25-BV421, CD27-BV205, CD38-APC, IgD-BB515 and IgG1k-BV421 isotype (Becton-Dickinson, Oxford, UK) and cells were analysed by flow cytometry. Memory B cells were identified as CD19^+^/CD27^+^ lymphocytes. The presence of class-switched (CD19^+^/CD27^+^/IgD^−^), unswitched (CD19^+^/CD27^+^/IgD^+^) and interleukin-2 receptor expressing (CD19^+^/CD25^+^/CD27^+^) memory B cell subsets was assessed. The number of immature (CD10^+^/CD19^+^/CD27^−^/CD38^+^); naïve/mature (CD10^−^/CD19^+^/CD27^−^/CD38^+^) and plasmablasts (CD19^+^/CD27^+^/CD38^+^) were estimated. The absolute number of cell subsets was calculated based on automated full blood counts (Sysmex XE-2100, Kobe).

### Cytotoxicity assay

Anonymised healthy donor blood samples were purchased from Cambridge Bioscience UK and mononuclear cells extracted. These were incubated with various concentrations of cladribine with or without 250 μM deoxycytidine to neutralise DCK activity [20]. Cells were stained with CD3-PE, CD19-PECy5 and CD27-BV205, Annexin V (Invitrogen, Loughborough, UK) and 4′,6-diamidino-2-phenylindole (DAPI) to detect live (Annexin V−, DAPI−), early (Annexin V+ , DAPI−) and late stage (Annexin V+, DAPI+) apoptosis. Cells were analysed using flow cytometry.

### B cell gene expression profiling

Gene expression of T cell and notably B cell subsets of *ADA*, *DCK* and *NT5*-related genes were identified through searches in public databases: at the Human Protein Atlas for immunohistology (http://www.proteinatlas.org, [21]) and Microarray and RNA*seq* data at BioGPS (http://www.biogps.org, [22]) and the Gene Expression Omnibus at the National Center for Biotechnology Information, Bethesda, USA (https://www.ncbi.nlm.nih.gov, GEO profiles/DATA sets).

### Statistical analysis

Sample size calculations were based on data within the CARE-MS I alemtuzumab trial data set [18], with 80% power to detect an 80% memory B cell depletion, comparable with the 12-month alemtuzumab depletion data [18], at the *P* = 0.05 (*n* = 4/arm) and *P* = 0.01 (*n* = 8/arm) levels. Relative proportions of B populations and live/apoptotic cells were determined using FlowJo 8 (FlowJo, Ashland, USA) software. Analysis of variance with Bonferroni post hoc test or Kruskal–Wallis analysis of variance with a Dunn’s post hoc test was performed using Sigmaplot software.

## Results

### Cladribine kills lymphocytes

**Fig. 1:**
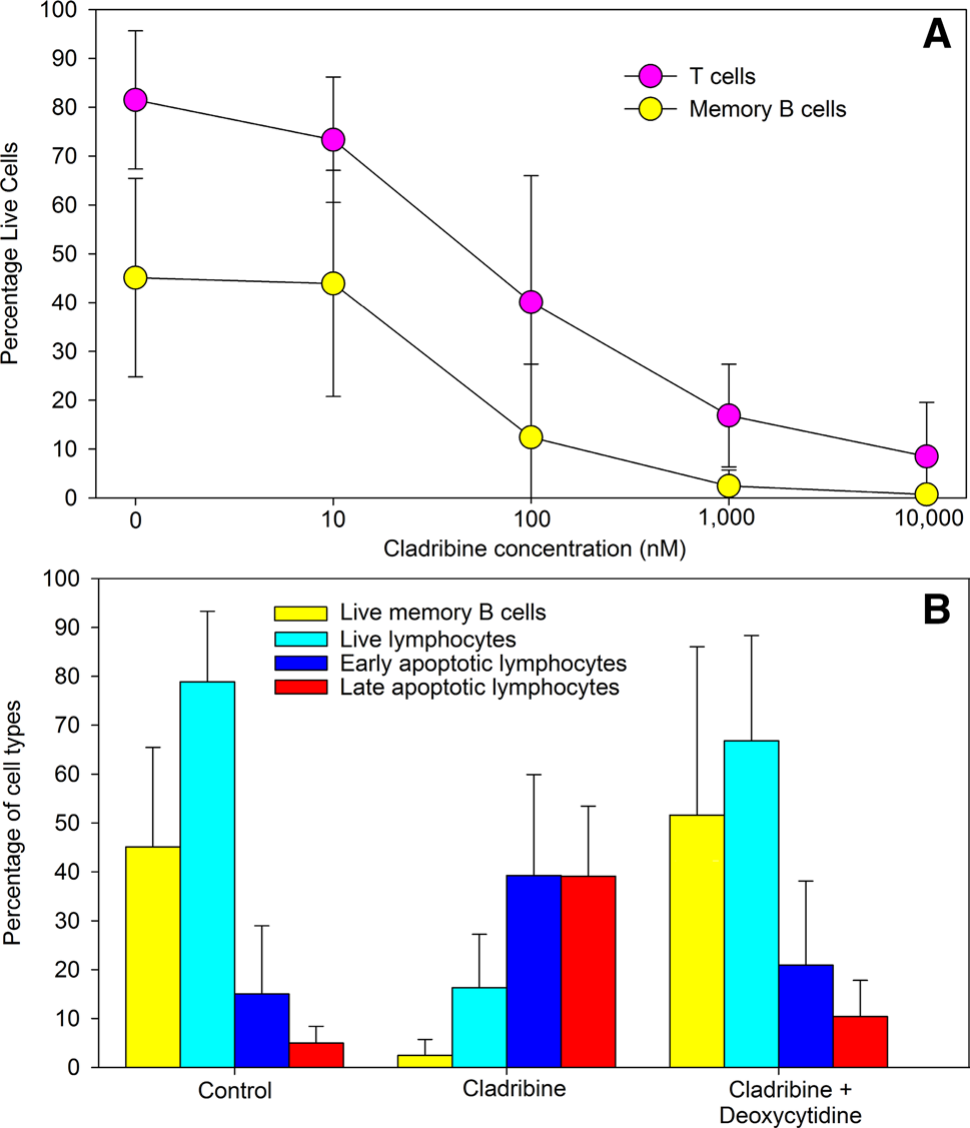
Cladribine induced lymphocyte killing in vitro. Peripheral blood mononuclear cells were incubated with various concentrations of cladribine for 70 h and were stained with Annexin V and DAPI to detect apoptotic and live cells and were phenotyped with CD3, CD19 and CD27 immunofluorescence using flow cytometry. **a** Cell viability and different cell subtypes and **b** Inhibition of 1 μM cladribine-induced lymphocyte cytotoxicity (Live = annexin V−, DAPI−; early = Annexin V+ , DAPI−; Late apoptosis = Annexin V+, DAPI+) in the presence or absence of 250 μM deoxycytidine. Results represent the mean ± standard deviation, *n* = 6

Analysis of the in vitro effect of cladribine demonstrated that healthy human lymphocytes were dose-dependently killed by cladribine (Fig. 1a). This occurred by apoptosis and was blocked by excess deoxycytidine (Fig. 1b), supporting DCK as a major mediator of the cytotoxic effect. Memory B cells were sensitive to cladribine, similar to T cells (Fig. 1a), at concentrations achievable in plasma in humans [20]. However, as anticipated, without additional activating stimuli, B cells rapidly died in culture even in the absence of cladribine, limiting detection of any possible influence for different immune subsets. Therefore, we examined the in vivo response to cladribine in MS.

### Cladribine induces limited severe lymphopenia compared to alemtuzumab

**Fig. 2:**
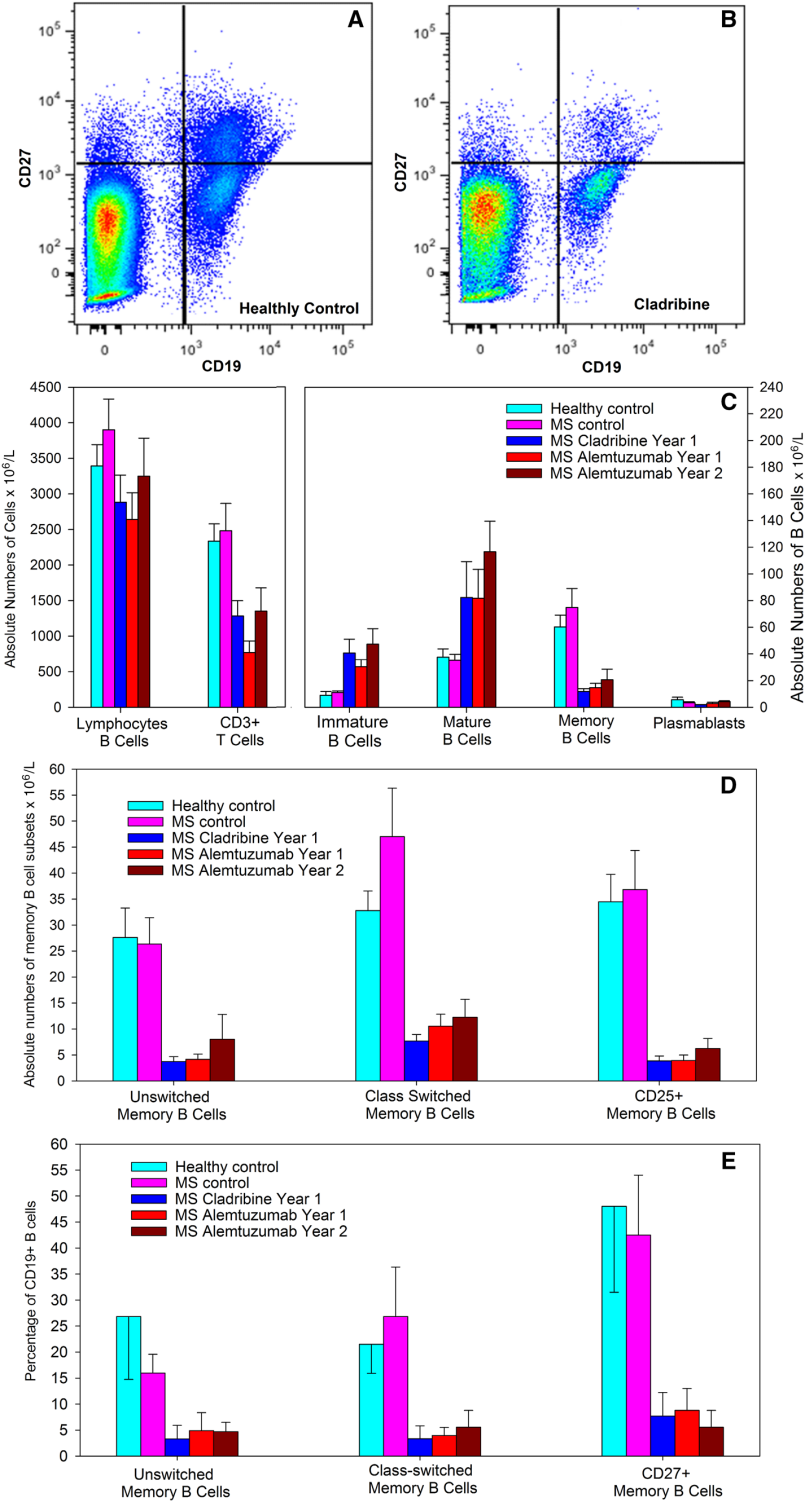
Cladribine induces marked memory B cells depletion. People with MS were treated with either subcutaneous cladribine of alemtuzumab and blood samples were taken at the last blood-screen prior to retreatment at year 1 (cladribine and alemtuzumab year 1) and 12 months after the second cycle (alemtuzumab year 2). Cells were stained with T and B cell markers assessed by flow cytometry and the results represent the mean ± standard deviation (*n* = 8/group). Flow cytometry of CD19 and CD27 staining in **a** a healthy control and **b** a person with MS treated with cladribine. **c** Total numbers of (left panel) T cells and (right panel) B cell subsets, **d** total numbers and **e** percentage of CD19^+^ B cells of immunoglobulin class-switched (IgD^−^) and unswitched (IgD^+^) and interleukin 2 receptor expressing memory B cells CD19^+^, CD27^+^ memory B cells

Our dose-adapted, subcutaneous cladribine induced a low (1.8%. *n* = 1/57) incidence of severe (grade 3/4) lymphopenia during the first cycle of treatment (Table 1). This was marginally lower than the 6.2% (*n* = 12/194) found in the immunophenotyping dataset of people taking oral cladribine (Table 1). Importantly the incidence of severe lymphopenia induced by subcutaneous cladribine was significantly (*p* < 0.0001) lower than the marked (84.1%, *n* = 106/126) lymphopenia induced by the first cycle of alemtuzumab treatment (Table 1). However, there was lymphocyte recovery towards the end of year 1 (Fig. 2).

**Table 1:** Lymphopenia induced by cladribine and alemtuzumab

Lymphopenia grade	Frequency/number of lymphopenic events at baseline and 1–2 months post-treatment		
	Oral cladribine (Mavenclad™)	Cladribine (Litak™)	Alemtuzumab (Lemtrada™)
Total	194 (100%)	57 (100%)	126 (100%)
Grade 0 (≥ lower limit of normal)	(95.4%) 185/89 (45.9%)	(98.2%) 56/31 (54.4%)	(96.8%) 122/1 (0.8%)
Grade 1 (< 1.0–0.8 × 10^9^/L)	(3.1%) 6/42 (21.6%)	(0.0%) 0/9 (15.8%)	(2.4%) 3/2 (1.6%)
Grade 2 (< 0.8–0.5 × 10^9^/L)	(1.5%) 3/51 (26.3%)	(0.0%) 0/16 (28.1%)	(0.8%) 1/17 (13.5%)
Grade 3 (< 0.5–0.2 × 10^9^/L)	(0.0%) 0/12 (6.2%)	(0.0%) 0/1 (1.8%)	(0.0%) 0/64 (50.8%)
Grade 4 (< 0.2 × 10^9^/L)	(0.0%) 0/0 (0.0%)	(1.8%) 1/0 (0.0%)	(0.0%) 0/42 (33.3%)
Frequency grade 3/4 lymphopenia	12/194 (6.2%)	1/57 (1.8%)	106/126 (84.1%)
Source	EMA report	Audit BartsHealth	Audit BartsHealth

Frequency of lymphopenia at baseline and up to 9 weeks after oral or subcutaneous cladribine or 4 weeks after alemtuzumab. These were assessed based on data from the EMA regulatory submission of the immunophenotyped cohort [16]. The other data was from anonymous audits of real-life use of subcutaneous cladribine and alemtuzumab. Lymphocyte levels were graded to reflect the degree of lymphopenia using the National Cancer Institute criteria

### Cladribine depletes peripheral blood memory B cells

Although serial analysis of B memory cell numbers following cladribine is needed and will be undertaken as a consequence of this study, initial analysis of cladribine-treated PwMS (*n* = 11) indicated that switched and unswitched memory B cell were consistently depleted to levels below reference levels [23] across a wide time-range (Supplementary Figure S1A). This suggests that once depleted, levels of B memory cells remain persistently low for over 12 months, as found in alemtuzumab- and rituximab-treated PwMS [18, 24]. Therefore, the memory B response was examined at a single point at the end of the treatment cycle in additional individuals (Figure S1B, Fig. 2a–d).

Healthy controls did not significantly differ from MS controls for any outcome measure assessed. However, there were significant treatment effects of both cladribine and alemtuzumab (Fig. 2a–e). There was depletion in the absolute numbers of CD3^+^ T cells in people treated with cladribine (45.0% depletion) and alemtuzumab (67.1%) at year 1 (Fig. 2c), which are similar with results of serial blood samples from the CLARITY [16] and CARE-MS I [18] studies. These pivotal phase III trials indicated that there was a marked depletion of CD19^+^ B cells by both alemtuzumab and cladribine [16, 18]. Consistent with the repopulation kinetics of alemtuzumab and rituximab [18, 24], there was an increase in the absolute numbers of immature and mature cells in PwMS treated with cladribine (Fig. 2c). These were comparable with the absolute B cell numbers in alemtuzumab-treated individuals (Fig. 2c). Most importantly, there was a marked (80.3%) and significant (*P* = 0.001) depletion of memory B cells by cladribine, compared to healthy controls, to levels at least as low to that seen in year 1 (75.6%) and year 2 (65.8%) samples post-alemtuzumab treatment (Fig. 2c).

Similarly there was a marked reduction (76.5 and 86.5% depletion compared to healthy controls) in the absolute numbers of both class-switched (*P* < 0.001) and unswitched (*P* = 0.003) memory B cells, respectively (Fig. 2d). This was comparable (67.8 and 84.9% depletion compared to healthy controls) to the class-switched and unswitched memory B cell depletion seen 1 year after the initial alemtuzumab treatment, respectively. Interestingly, there was also selective depletion of the CD25^+^ subset of memory B cells (Fig. 2d). These cells represented 63.3 ± 4.3% of the CD19^+^, CD27^+^ pool in healthy controls, but only 34.1 ± 2.0% of the memory B cell pool in PwMS treated with cladribine and 27.0 ± 3.6% (year 1) and 32.4 ± 4.4% (year 2) in PwMS treated with alemtuzumab, indicating a marked depletion in this subset by cladribine (88.9% depletion compared to healthy controls) and alemtuzumab (88.6% depletion at year 1, Fig. 2d).

To aid comparison with other published studies [4, 19], the percentage of total B memory cells within the CD19^+^ population (Figure S1B and 2E) was also analysed. Both class-switched and unswitched memory B cells were significantly (*P* < 0.05) reduced following cladribine treatment to a level comparable with that found following alemtuzumab treatment at year 1 and year 2 (Fig. 2d).

### Expression of purine salvage pathway genes suggests selective vulnerability of B cells to cladribine

**Fig. 3:**
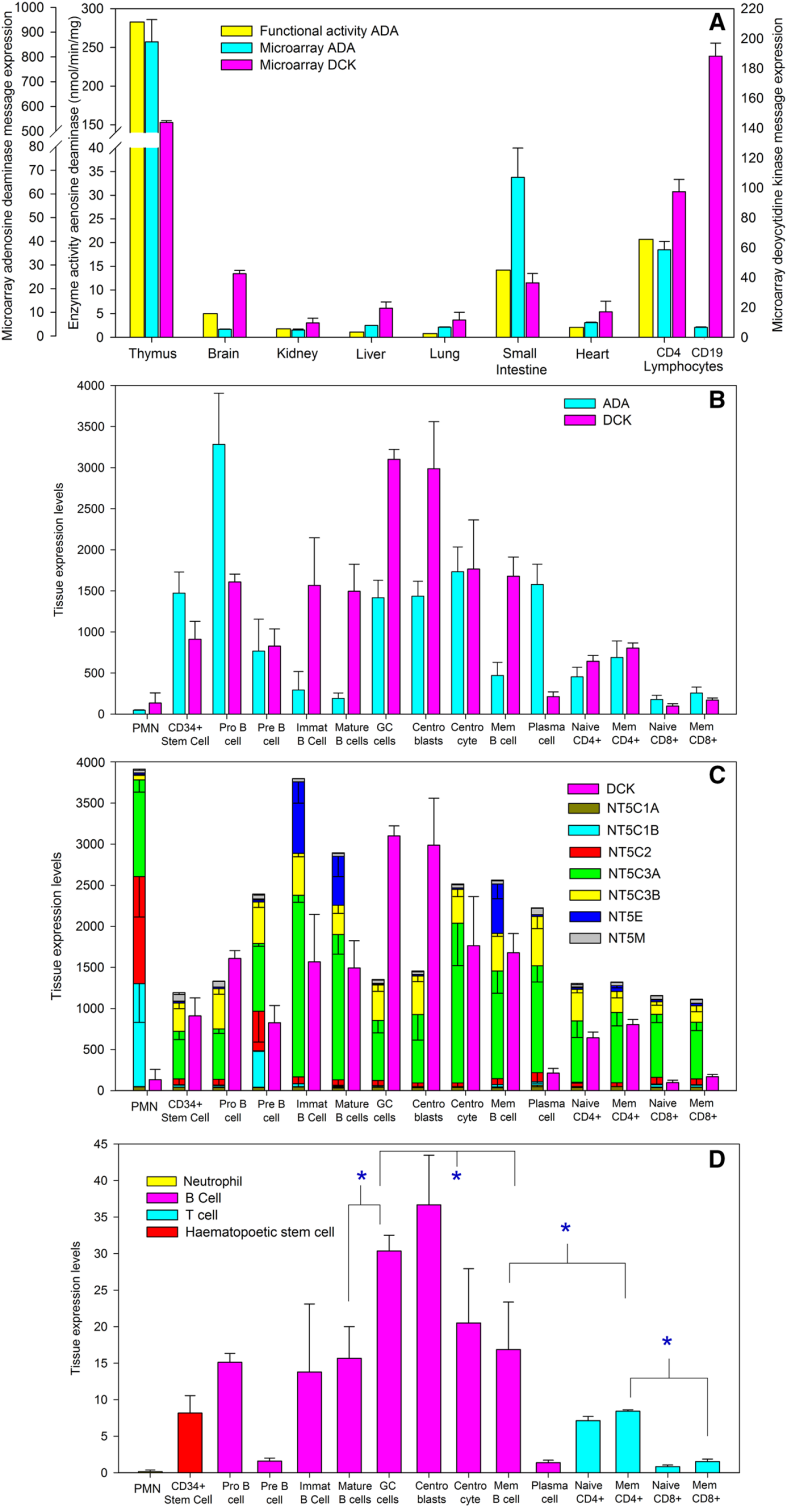
Microarray expression of purine salvage pathway genes indicates a B cell sensitivity to cladribine. Publically available microarray expression data (http://www.biogps.org) was extracted from the **a** Geneatlas U133, gcrma and **b**–**d** Primary cell Atlas. DBS_00013. **a** Microarray detected gene expression of adenosine deaminase (ADA. 204639_at) and deoxycytidine kinase (DCK. 203303_at) in various tissues in the Geneatlas U133, gcrma. Identifier GSE1133 (http://www.biogps.org). The results represent the mean ± SD in duplicate samples. This was compared to the distribution of function protein expression reported previously [14]. **b**–**d** The data represent the mean ± SD expression Z scores from: neutrophils (*n* = 4), CD34^+^ hematopoietic stem cells (*n* = 6), Pro-B (*n* = 2), Pre B (*n* = 2), immature B cells (Immat, *n* = 3) and tonsillar mature cells (*n* = 3), germinal centre cells (GC cells, *n* = 4), centroblasts (*n* = 4), centrocytes (*n* = 4), memory B cells (mem, *n* = 3) and plasma cells (*n* = 3), naïve and effector memory (Mem, CCR7^−^, CD45RO^+^) CD4^+^ (*n* = 5/group) and CD8^+^ T cells (*n* = 4/group). The expression of **a** ADA (204639_at) and DCK (23302_at). **b** The expression of DCK and 5′NT detecting by: NT5C1A (224549_s_at), NT5C1B (243100_at), NT5C2 (209155_s), NT5C3A (225044_at), NT5C3B (209155_s_at), NT5E (203939_at) and NT5M (219708_at). **c** Expression ratio of DCK expression divided by expression score of NT5C1A and NT5C1B 5′NT that can dephosphorylate adenosine/monophosphate. *Significantly different between groups (*P* < 0.05)

**Fig. 4:**
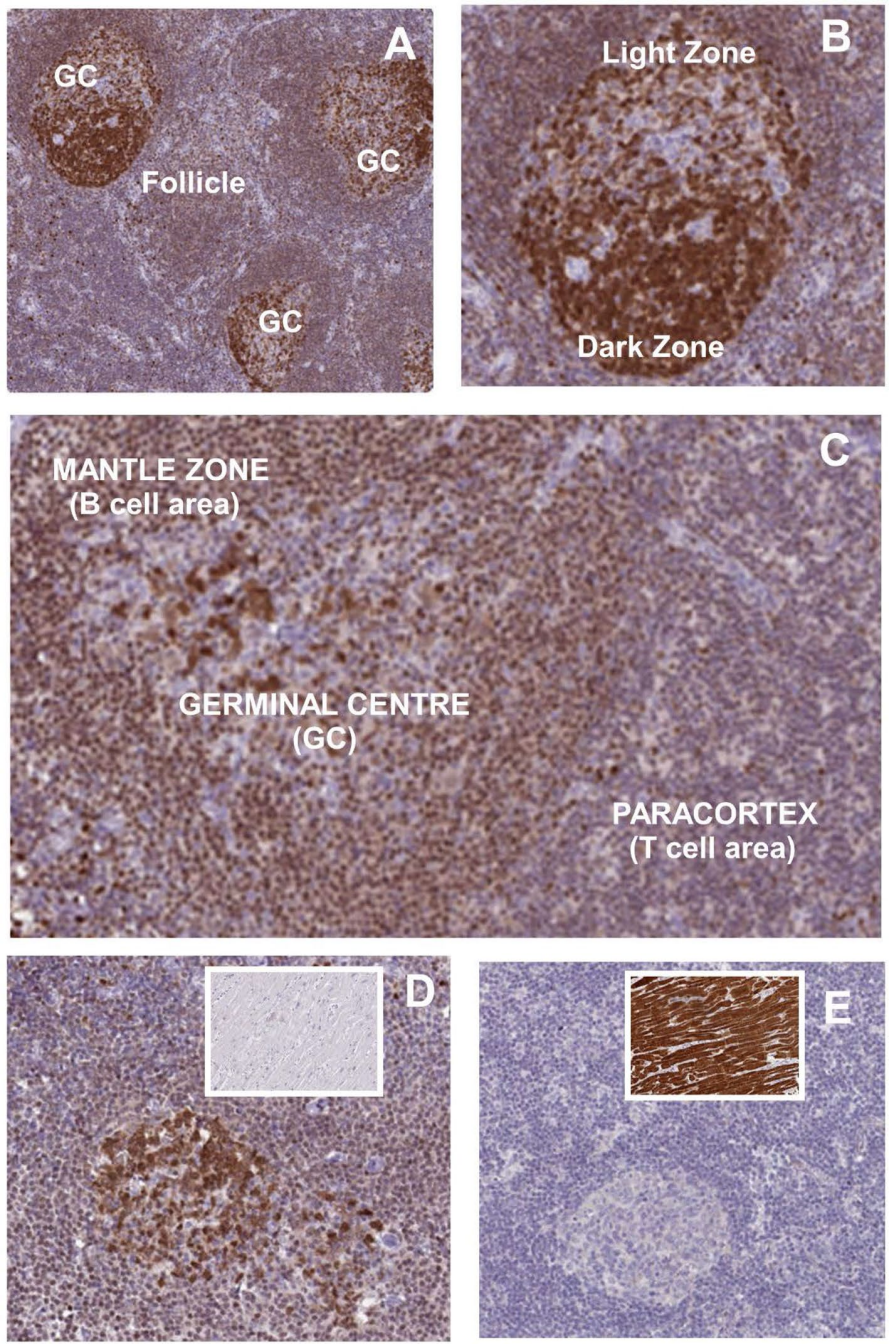
Deoxycytidine kinase expression in lymph node tissue. DCK expression was immunostained in three (**a**–**d**) people showing: **a** DCK expression in follicle and secondary follicles containing high-intensity staining in the germinal centres (GC). **b** Intense immunostaining was present notably in the germinal centre B cells and centroblasts in the dark zone, compared to the light zone contains centrocytes, memory B cells and plasma cells. **c** Enhanced staining in secondary follicles compared to the paracortex. **d** DCK expression in lymph nodes with heart myocyte staining in the inset. **e** NT5C1A expression in lymph nodes with heart myocyte staining in the inset. Elevated message was detected in skeletal and heart muscle compared to peripheral blood mononuclear cells in GEO identifier GDS 3113. Immunostaining is reproduced under the Creative Commons Attribution-Share Alike 4.0 international licence. Images, including description of tissue donor, available from http://V17.proteinatlas.org, https://www.proteinatlas.org/ENSG00000116981-NT5C1A/tissue/lymph+node#img, https://www.proteinatlas.org/ENSG00000116981-NT5C1A/tissue/heart+muscle#img, https://www.proteinatlas.org/ENSG00000156136-DCK/tissue/lymph+node#img and https://www.proteinatlas.org/ENSG00000156136-DCK/tissue/heart+muscle

Gene and protein expression data within public repositories were searched to determine whether differences in purine salvage pathway genes could help explain selectivity of the action of cladribine for B cells. It was evident that ADA and DCK are relatively specific for leucocytes in contrast to nucleoside phosphorylases that have broad expression [13]. This was readily observed following examination of microarray data (Fig. 3a, b). It was found that the tissue distribution of *ADA* message correlated well with the previously reported [13] protein activity (Fig. 3a). Furthermore, although there was variation in lymphocyte expression levels between different microarray studies, it was evident that B cells often express lower levels of ADA than T cells (Fig. 3a, b, E-GEOD-22886, GSE62584 from blood during first demyelinating event) and importantly B cells may, but not always (E-GEOD-22886, GSE62584), express higher levels of DCK than T cells (Figs. 3a, b, 4). This is consistent with observations measuring protein or functional activity of the enzymes within normal cells and malignant cells, where B lineage cells tend to exhibit higher activity than T lineage cells [25]. However, it was evident that B cell subsets are very heterogeneous with regard to expression (Fig. 3b). Whilst there was variation between different microarray studies (GPS_00013; E-GEOD-22886; GSE68878; GSE68245; GSE68878) on balance it was found that immature, mature and memory populations, which populate the blood compartment, had similar levels of DCK (Fig. 3b). These expressed low levels of ADA (Fig. 3b). However, it was consistently found (GPR_00013; GSE68878; E-GEOD-22886) that plasma cells in blood, tonsil and bone marrow (Fig. 3b) exhibited significantly lower levels of DCK compared to memory and germinal centre cells. Interestingly, it was evident that germinal centre cells and notably lymphoblasts, which localise to the dark zone of the germinal centre exhibit high levels of DCK (Fig. 3b, E-GEOD-38697; E-GEOD-15271). This profile was consistent with protein expression within human lymphoid tissue (Fig. 4). Indeed B cells within the follicles express more staining than cells within the paracortical areas, which contain T cells (Fig. 4a–d). Importantly there was high expression of DCK within the dark zone of the secondary follicles (Fig. 4a–d). Within the light zone there were intensely stained, modestly stained and poorly stained cells, which is perhaps consistent with levels of DCK message in centrocytes, memory cells and plasma cells (Fig. 3b) that reside in these areas.

Although DCK is expressed at high levels within B cells, some 5′NT subtypes, notably NT5C3 isoforms are expressed at high levels within B cells (Fig. 3c). This could counter the action of DCK. Likewise, NT5E was expressed at higher levels in the circulatory: immature, mature and memory subtypes than T cells (Fig. 3c), which is consistent with reports on protein expression [25]. Whilst NT5E will dephosphorylate deoxyadenosine monophosphates, as it is an ecto-enzyme, it may not be particularly relevant to control the cytosolic action of DCK [26]. Likewise, as cytosolic NT5C2 preferentially targets inosines and NT5C3 isoforms are involved in pyrimidine catabolism [26], their relevance to dephosphorylation of 2-deoxycladribine monophosphate remains to be properly identified. However, based on the ratio of DCK activity to total 5′NT activity, the germinal centre cells would appear to be a potentially highly sensitive to cladribine (Fig. 3d). This aspect is particularly notable, when the balance between DCK and the cytosolic adenosine monophosphate degradative NT5C1 isoforms [26] was examined (Fig. 3d). These are expressed at low levels (Figs. 3c, 4e). There was a significantly higher DCK:NT5C1 ratio in CD4 T cells than CD8 T cells and higher DCK:NT5C1 ratio between mature and memory B cells and memory CD4^+^ and CD8^+^ T cells (Fig. 3d). In contrast neutrophils expressed little DCK message and more NT5C1B (Fig. 3d). This may help explain the pattern of neutrophil, T cell and B cell depletion profiles observed following use of cladribine.

## Discussion

Although memory B cells have previously been shown to be suppressed by alemtuzumab in MS, with numbers remaining low for at least 50 weeks post-therapy [18], this action was extended here to show an effect on activated and both immunoglobulin class-switched and unswitched memory B cells. Importantly, to our knowledge this is the first evidence of the effect of cladribine on the memory B cell pool. These data support the hypothesis that memory B cells play an important role in the pathogenesis of MS [4]. This data is consistent with the observation that agents that inhibit relapsing MS, with the exception of natalizumab that increases peripheral blood memory B cells presumably because they are prevented from entering the CNS, deplete peripheral memory B cells in a manner that appears to reflect their level of treatment efficacy [4, 19]. Importantly, if memory cells are central to the action of DMT, that cladribine and alemtuzumab induced comparable memory B cell depletion, at least at about 1 year after treatment, indicates that the personalised, adaptive-dosing of generic cladribine can limit the occurrence and potential consequences of severe lymphopenia, possibly without a potential detriment to efficacy. However, serial monitoring of peripheral memory B cells will be required to determine the extent and longevity of the response. There is a lack of correlation of MS activity with the level of T cell subset deletion [19]. Therefore, it will be of interest to determine whether peripheral blood memory B cell levels correlate with lesional/clinical activity, where CD8 T cells and memory B cells accumulate [27], as seen in some other CD20-sensitive autoimmune conditions [28–30].

The results obtained with alemtuzumab are comparable with results from the CARE-MS I study [18]. It will be important to determine the extent of memory B cell depletion following oral cladribine use. However, it is anticipated that it may be similar to this study as comparable amounts of cladribine was administered based on the 40% bioavailability of the oral formulation compared to 100% bioavailability of the subcutaneous formulation used here [31]. Indeed the level of lymphocyte, CD3 and CD19 T and B depletion by subcutaneous depletion observed here is similar with results of serial blood samples from the CLARITY oral-cladribine study [16]. Although, we did not measure T cell subset depletion in this study, the level of CD3 depletion seen here (45.0% depletion in the mean number of CD3 cells in PwMS treated with cladribine at 44 weeks compared to controls) with subcutaneous cladribine was similar to that reported previously with 3.5 mg/kg oral cladribine (39.1 ± 2.4% depletion at week 44 from baseline), where the absolute number of CD4, CD45R0^+^ memory T cells was depleted by 34.4 ± 6.3% (at 9 weeks) and 37.0 ± 2.9% (at 44 weeks) from baseline [16] and CD8, CD45RO^+^ memory T cells were depleted by only 15.2 ± 2.5% (at week 9) and 13.5 ± 6.3% (at week 44) from baseline. This may suggest that depletion of these T subsets by the dose of subcutaneous cladribine used here, may be limited also, but further studies are warranted to include analysis of Th1, Th17 cells and T regulatory cells to determine whether selective depletion occurs following cladribine. Indeed, by increasing the dose and frequency of dosing, more substantial depletion T cell, notably CD4 T cell depletion, can be achieved [16, 17]. This may contribute to and even account for the clinical efficacy observed [7, 8]. This study does not prove that memory B cells actually mediate an essential component of relapsing MS. Likewise, definitive proof that T cells are the central mediators of the action of DMT in MS in humans is circumstantial and unproven also and the levels of their peripheral blood depletion do not correlate with efficacy [4, 16]. However, it is evident that DMT have a number of possible mechanisms of action [4] and given the activity of CD20-depleting antibodies [6], it is essential that the importance of B cells in disease control is considered.

The relatively high B cell expression levels of DCK, coupled with low levels of NT5C1 and ADA, compared to T cells, and neutrophils, and the higher turnover of B cells may create the B cell-selective depleting effect of cladribine seen in vivo [16, 32]. This effect coupled with the slow kinetics of memory B cell repopulation [18, 23], may explain why memory B populations are vulnerable to cladribine. As seen with other DMT including rituximab and alemtuzumab [18, 24], the peripheral blood mature B cell population expands following depletion from the immature B cell pool that rapidly enter the blood, probably from the bone marrow [18, 24]. This probably accounts for the apparent rapid normalisation of CD19^+^ B cells that reconstitute the blood faster than T cells [5, 16, 18]. It has been recently questioned whether fingolimod inhibits the action of alemtuzumab due to sequestration of immune cells within lymphoid tissue, probably the bone marrow, which may not be effectively purged by alemtuzumab [33]. This could perhaps account for the rapid hyper-proliferation of immature and mature B cells seen after alemtuzumab treatment [18]. The slower repopulation of B cells may reflect the fact that bone marrow, B cell precursor cells have deoxycytidine kinase and should therefore be sensitive to depletion by cladribine. Cladribine therefore behaves as a chemical CD19-depleter. In contrast to immature and mature cells, peripheral blood memory B cells repopulate very slowly from the lymphoid organ pool mostly via germinal centre activity [34]. The germinal centres may be particularly vulnerable to inhibition by cladribine due to the high level of proliferation and DCK expression, leading to selective long-term loss of peripheral blood memory B cells. These may be depleted for 18 months or substantially longer in some individuals, as seen in development [27] and following depletion with alemtuzumab or rituximab [18, 28]. As the expression of DCK in plasma cells was low, it suggests that long-lived plasma cells may be relatively unaffected as occurs following CD20^+^ B cell depletion, and thus not interfering with pre-existing vaccination responses. However, this remains to be established.

The longevity of the memory B cell depletion may be a key contributor to the mechanism of “induction therapy” activity of these pulsed immune-reconstitution treatments. Pathogenic memory cell repopulation may be a simple reason for treatment failures, requiring re-dosing, which occurs with cladribine and alemtuzumab [5, 7–9]. Furthermore, it remains to be established whether the peripheral memory B cell compartment has value as a biomarker for response to disease activity, therapy and as an indicator for retreatment, as occurs in a number of other neurological and non-neurological, CD20 depletion-sensitive autoimmune conditions [28–30].

Although we have placed emphasis on the potential role of the memory B cell, the thymus is targeted by cladribine [35] and T cells still harbour elevated DCK levels, compared to other tissues, and are depleted. Furthermore, the action of cladribine may block the antigen presentation function of memory B cells and thus silence the remaining autoreactive T cells, using mechanisms suggested for CD20-depleting antibodies [4]. Therefore, cladribine may control MS via an action on both T and B lymphocytes, notably as T cell activities are integrally involved in B cell function. However, the importance of memory B cells is consistent with the potential genetic and infectious aetiology of MS [36]; the pathology that is associated with memory B cell accumulation during attacks [27, 37], the generation of nerve and oligodendrocyte cytotoxic molecules and ectopic B cell follicle formation [4, 27, 38]; and importantly the response to effective DMT [4]. Epstein Barr Virus is believed to be an aetiological trigger of MS and drives the production of memory B cells, which may become relatively T cell-independent, and perhaps more autoimmune prone, due to B the antigen receptor and CD40-signalling mimics created by the virus [36, 39].

The data presented here indicates that cladribine depletes memory B cells, in addition to T cells, and suggest that memory B cells could possibly be useful targets for the development of new, specific and better tolerated drugs for MS treatment. Also, in the shorter term, it is possible that we will be able to improve effectiveness and patient safety by routinely monitoring memory B cell levels. The oral and subcutaneous routes produce cladribine with defined pharmacokinetics that allow bioequivalent doses to be selected [31]. Our subcutaneous cladribine protocol was originally developed for compassionate use for people without access to treatment, rather than an alternative to the commercial product. As such it has only been administered to PwMS from a few local centres within the UK. This may change as our experience becomes published and neurologists and nurses become comfortable using the oral formulation that has been licensed and is available for use in highly active relapsing MS within the UK and the rest of Europe [10]. This could lead to the development of treatment options for all people with MS.

## Electronic supplementary material

Below is the link to the electronic supplementary material.

Supplementary material 1 (DOCX 635 kb)

## Abbreviations

5′NT: 5′ Nucleotidase
ADA: Adenosine deaminase
DCK: Deoxycytidine kinase
DMT: Disease-modifying treatments
EDSS: Extended disability status scale
EMA: European Medicines Agency
MS: Multiple sclerosis
PwMS: People with MS
