# Supplementary material for: Cladribine treatment of multiple sclerosis is associated with depletion of memory B cells
Source: J Neurol. 2018 Mar 17;265(5):1199–209. doi: 10.1007/s00415-018-8830-y (PMC5937883; doi:10.1007/s00415-018-8830-y)
Supplement: Supplementary file 1 — Supplementary material 1 (DOCX 635 kb) [file 415_2018_8830_MOESM1_ESM.docx]

**SUPPLEMENTARY TABLE S1**

*Demographics of People enrolled in the immunophenotyping study*

| Group | Sex (M/F) | Age (Yr) | Disease duration (Yr) | WebEDSS Median (Range) |
| --- | --- | --- | --- | --- |
| Healthy controls | 5/4 | 36.1 ± 9.9 | N/A | N/A |
| MS controls | 2/6 | 36.7 ± 8.2 | 3.9 ± 4.2 | 3.0 (0.0-5.0) |
| Cladribine | 2/6 | 46.8 ± 11.4 | 6.3 ± 3.1 | 5.25 (0.0-6.5) |
| Alemtuzumab year 1 | 2/6 | 38.0 ± 10.6 | 4.6 ± 2.4 | 3.0 (0.0-6.5) |
| Alemtuzumab year 2 | 3/5 | 40.3 ± 8.2 | 5.8 ± 3.9 | 3.0 (0.0-5.0) |

Demographic analysis of PwMS enrolling in the phenotyping study. The results represent: the number of people within each gender; The mean ± SD age of the participants; The mean ± SD duration of disease from first symptom. The median and range WebEDSS (https://edss.clinicspeak.com) score. All people had a diagnosis of relapsing MS, except 3 people in the cladribine treated group who were considered to have advanced (progressive) MS. Analysis of variance revealed no significant differences between of people with MS groups and no differences in ages.

**SUPPLEMENTARY FIGURE 2**.


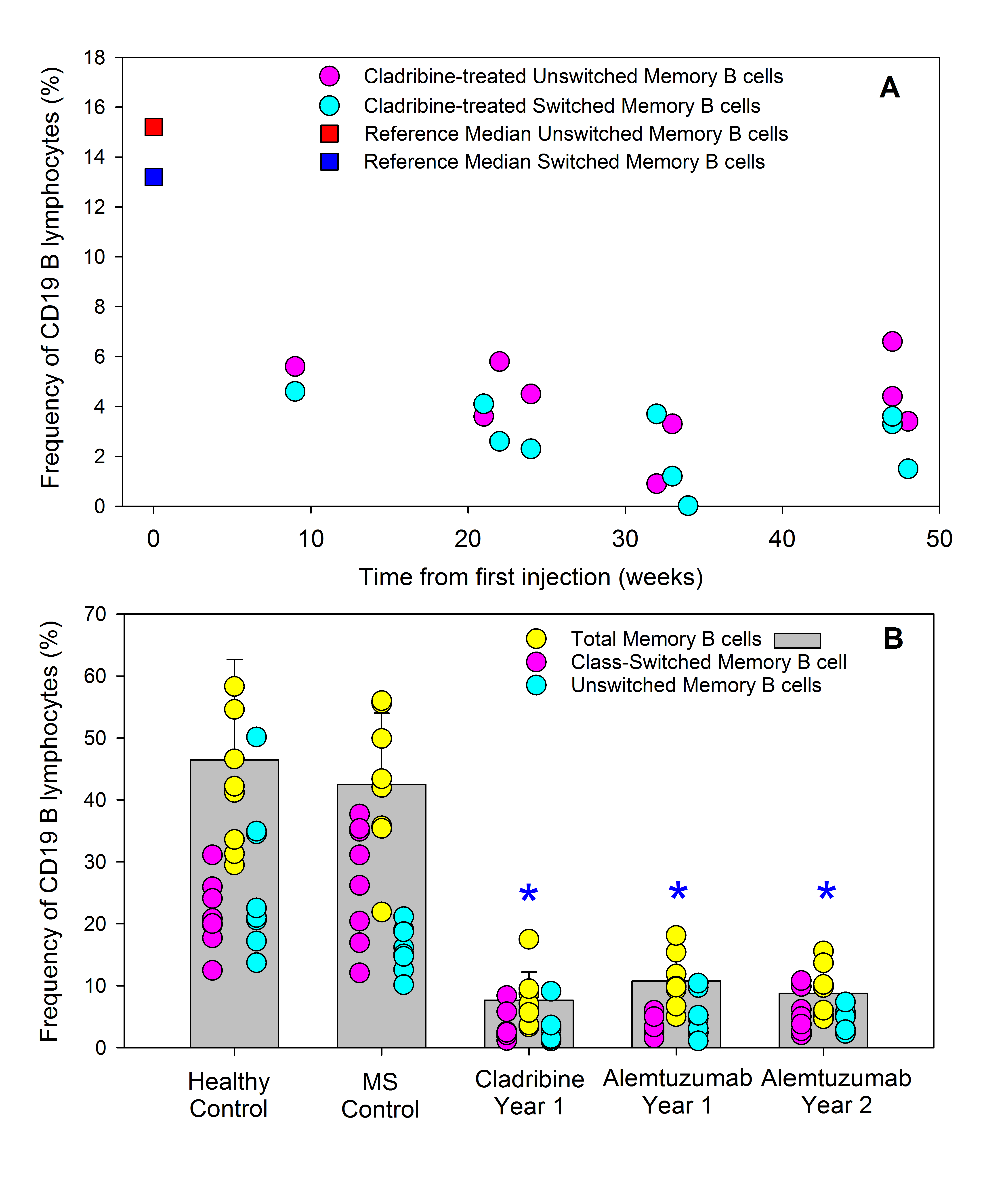


*Frequency of class-switched and unswitched memory B cells following subcutaneous cladribine administration.* People with MS were treated with subcutaneous cladribine (Total dose 40mg-60mg) on week 1 and again on week 5. Blood samples were taken: (**A**) at various times during the first year of treatment n=11. The reference range of B cells was reported previously [24]. Values from control samples can be seen in Figure 2B. (**B**) At the last blood-screen prior to retreatment at year 1 (cladribine and Alemtuzumab Year 1) and 12 months after the second cycle (Alemtuzumab Year 2). Cells were stained with CD19, CD27 and IgD specific antibodies. The results represent individual percentage number of immunoglobulin class-switched (CD19^+^, CD27^+^, IgD^-^) and unswitched (CD19^+^, CD27^+^, IgD^+^) memory cells of the CD19 population (n=8/group). *P<0.05 compared to healthy controls for all three populations.
